# A novel technique for visualizing *in vivo* dynamic neuronal changes during *Drosophila* development

**DOI:** 10.17912/micropub.biology.002359

**Published:** 2026-10-21

**Authors:** Natalie Rico Carvajal, Chris Q Doe, Kristen M Lee

**Affiliations:** 1 Institute of Neuroscience, University of Oregon, Eugene, OR, United States; 2 Howard Hughes Medical Institute, Chevy Chase, MD, United States

## Abstract

A major limitation in neurobiology is the inability to precisely track *in vivo* neuronal morphology over time. Tandem dimer Eos (TdEOS), a fluorescent photoconvertible protein under UV light, enables visualization and quantification of morphological changes between timepoints in a single animal. We optimized this using the *Drosophila* Moonwalker Descending Neuron (MDN), which undergoes morphological changes and is genetically accessible. By expressing TdEOS in MDNs, we demonstrated successful *in vivo* photoconversion, confocal imaging, and quantification of dendrite morphology changes between two timepoints in the same animal. Ultimately, TdEOS provides a targeted, accessible tool for tracing complex neuronal morphology overtime *in vivo* .

**Figure 1. TdEOS allows visualization of morphological changes during Drosophila MDN development:** **A)** Schematic showing the TdEOS method protocol. The flies are reared in the dark at 25°C. At 72 hours ALH, larvae are exposed to 405 nm UV light for 20 minutes. Half the larvae are dissected immediately after light exposure, while the other half are aged for an additional day (96 hours ALH). The dissected larval brains then undergo confocal imaging. **B)** Schematic of MDN photoconversion timeline and morphology. **C)** Schematic of quantification. **D)** 3D projection of microtubule TdEOS signal: MDN hemi-neurite labeled both green and magenta. White arrows show the extend of dendrite visualization and photoconversion. Scale bar, 10µm. **E)** 3D projection of myristoylated TdEOS: MDN hemi-neurite dissected at 72 hours ALH vs. 96 hours ALH. White dashed line outlining magenta hemi-neurite signals. Yellow arrows shows new growth. Scale bar, 10 µm. **F)** Areas of the MDN hemi-neurite green and magenta signals from animals dissected at 72 and 96 hours. **G)** Quantification of myristoylated TdEOS average area green to magenta ratio of the MDN dendrites at 72 hours (blue) and 96 hours (yellow) ALH. Green to magenta ratio statistics: t-test, p < 0.0001, n = 7-8 MDN hemi-neurite. **H)** Quantification of myristoylated TdEOS average area green minus magenta of the MDN dendrites at 72 hours (blue) and 96 hours (yellow) ALH. Green minus magenta statistics: t-test, p < 0.0001, n = 7-8 MDN hemi-neurites.

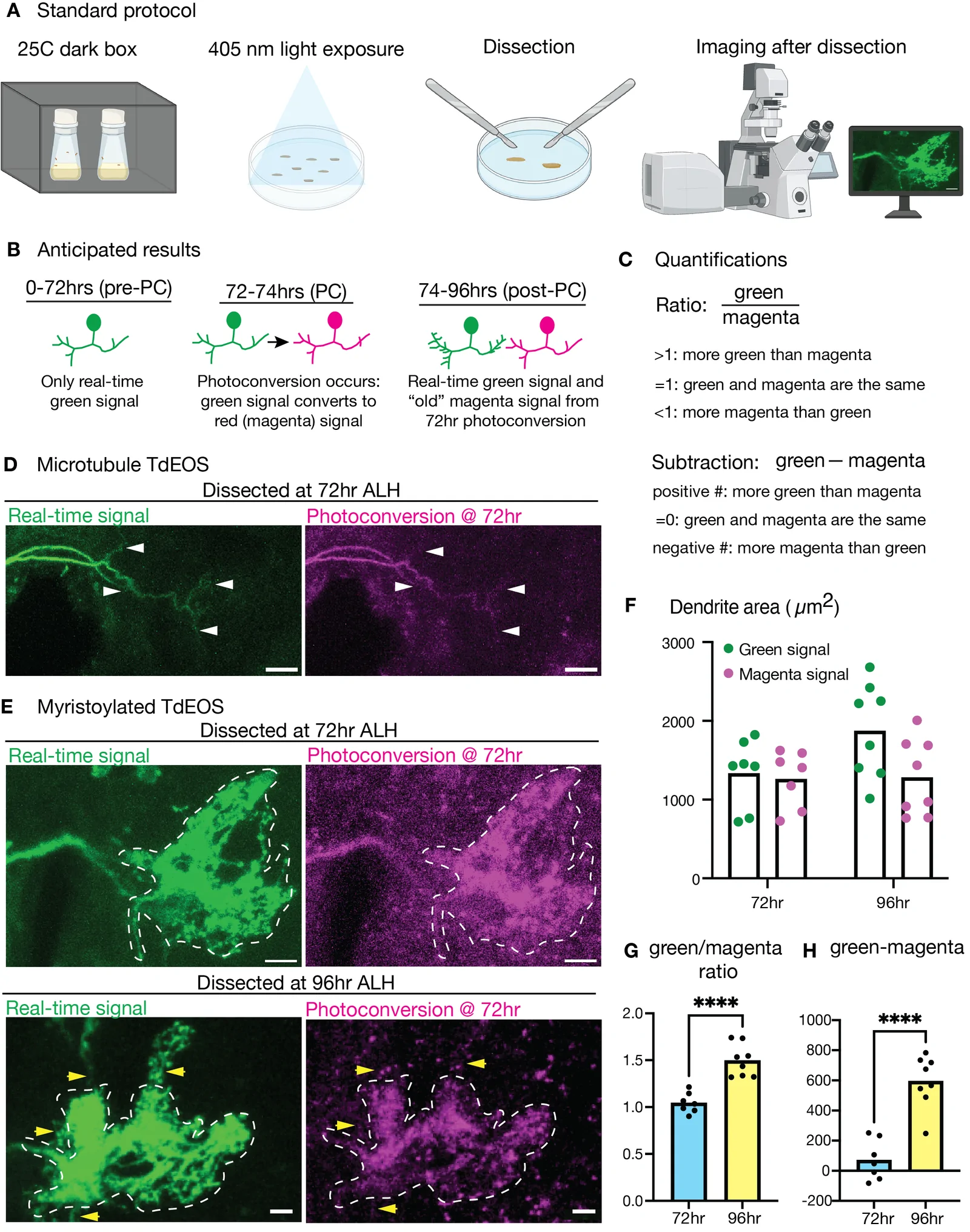



## Description

Established imaging techniques, including long-term live-cell imaging of standard fluorescent reporters like GFP or mCherry, have been helpful for visualizing basic neuroanatomy but require complicated setups for dynamic tracking of neuronal structures overtime, particularly *in vivo* . These specific hurdles are keeping the tissue alive and preventing photobleaching. Here, we present a targeted approach that allows long-term tracking of neuronal growth in *Drosophila melanogaster* , utilizing the tandem dimer Eos (TdEOS), a photoconvertible fluorescent protein, and a standard confocal microscope (Betzig et al., 2006; Wiedenmann et al., 2004). TdEOS irreversibly converts from green to red emission upon targeted exposure to 405 nm light – effectively taking a snapshot of the protein expression at one timepoint, while maintaining real-time expression of the green signal provided from new protein being made (Betzig et al., 2006; Wiedenmann et al., 2004). This provides a visual marker differentiating the UV light exposure time point from all other time points - enabling the strict temporal isolation of neuronal morphology at a specific developmental stage of interest while allowing comparisons to the real-time morphology in the same animal.

Photoconversion techniques are not novel in *Drosophila* , and have been proven to be highly useful. A popular example is Calcium-Modulated Photoactivatable Ratiometric Integrator (CaMPARI), which has been successfully used to label functional neuronal synaptic partners in the larvae and adults (Carreira-Rosario et al., 2018; Edwards et al., 2020; Lee et al., 2025; Moeyaert et al., 2018). The technique utilized here, TdEOS, has been used to study rapid dendritic growth in ddaC neuron dendrites, using live confocal imaging with larval and pupal dissections at 0 minutes and 30 minutes post-light exposure (Bu et al., 2021). Given that it is well-documented that neuronal morphology can change across an animal’s life, we hypothesized that this TdEOS technique could be further optimized to also track long-term morphology changes, in addition to rapid growth. To investigate this, we utilized the *Drosophila* Moonwalker Descending Neurons (MDNs) since they undergo morphology changes throughout life and are genetically accessible (Carreira-Rosario et al., 2018).

Our novel TdEOS technique is unique in its ability to easily view real-time dynamic changes in neuronal morphology at very different timepoints. Here, we optimized for 24 hours between the initial UV light exposure/photoconversion and standard confocal imaging ( Figure 1A, B). We expressed TdEOS specifically in the Moonwalker Descending Neurons (MDNs) using an MDN-GAL4 driver. When MDN expresses the TdEOS transgene, MDN is labeled with GFP (i.e. green signal), regardless of photoconversion occurring. Larvae were reared at room temperature (25 °C) and in the dark until 72 hours after larval hatching (ALH), when larvae were exposed to 405 nm UV light ( Figure 1A ), making MDN photoconvert to magenta (581 nm “red” signal pseudo colored magenta, henceforth referred to as magenta; Figure 1B ). After photoconversion, the larvae were sorted into two equal groups. Half of the larvae were immediately dissected and imaged after photoconversion at the 72-hour timepoint. The remaining half of the larvae were stored at room temperature on standard food and were dissected and imaged at the 96-hour timepoint. For all these animals, there are two signals labeling different versions of MDN: the magenta (photoconverted) signal is a snapshot of what MDN looks like at 72 hours ALH, while the green (non-photoconverted) signal is what MDN looks like in real-time ( Figure 1B ). We scored photoconversion using these signals, as no magenta signal meant the photoconversion did not occur; we only proceeded with animals that had both signals, which occurred over 80% of the time using our experimental setup.

Additionally, quantification of this data is simple because the comparisons are being made between two signals in the same animal. To start, we measured the area of the magenta signal (photoconverted) and green signal (non-photoconverted) ( Figure 1C ). We quantified this data two different ways. First, we divided the green average area of a MDN dendrite by the magenta average area of the same MDN dendrite. This gives us a ratio of “new” green (non-photoconverted) signal over “old” magenta (photoconverted) signal, meaning a ratio of greater than one demonstrates that there is more “new” MDN dendritic material at 96 hours ALH than the “old” MDN signal from the photoconversion at 72 hours ALH ( Figure 1C ). Alternatively, we subtracted the “new” green (non-photoconverted) signal MDN dendritic material area by the “old” magenta (photoconverted) signal area of the same MDN dendrite. For this quantification, a positive value demonstrates that there is more “new” MDN dendritic material at 96 hours ALH than the “old” MDN signal from the photoconversion at 72 hours ALH, indicating that growth occurred between the 405 nm light exposure and dissection timepoint ( Figure 1C ).

Next, we investigated how the MDN dendrites are changing between 72 and 96 hours ALH using the TdEOS experimental and quantification protocols described above ( Figure 1A- C), with a goal to study secondary and tertiary dendrite dynamics and volume over time. We used two different transgenic versions of TdEOS: a microtubule-tagged version and a myristoylated (myr; membrane) version. First, we used the microtubule::TdEOS transgene, but this TdEOS variant only labeled the primary neurite, with minimal expansion into the secondary or tertiary dendrites of MDN ( Figure 1D ). While interesting biologically, we moved forward experimentally with the myr::TdEOS transgene, which labels the whole plasma membrane and successfully labeled the entire volume of the MDN dendrites ( Figure 1E ). After imaging the 72hr and 96hr old animals, we quantified the areas of the green (non-photoconverted/ “new”) dendritic region and the magenta (photoconverted/ “old”) dendritic region ( Figure 1F ). As anticipated, at 72 hours ALH, immediately after UV light exposure, the MDN dendrites look the same in the photoconverted magenta and the non-photoconverted green signals ( Figure 1E, F). This demonstrates that photoconversion is occurring immediately after illumination. Importantly, in animals dissected at 96 hours ALH, the signal photoconverted at 72 hours ALH has a similar area to the animals that were dissected at 72 hours ALH ( Figure 1F ). This demonstrates that between 72 and 96 hours ALH, the photoconverted signal strength is maintained, and it retains the dendritic morphology present when the photoconversion occurred. Using these data, we asked whether MDNs dendrite morphology is changing between 72 and 96 hours ALH. At 96 hours ALH, the green non-photoconverted/real-time signal clearly extends outside of the domain of the magenta photoconverted signal ( Figure 1E ). In support of this, the green/magenta ratio was significantly increased at 96 hours compared to 72 hours ALH ( Figure 1G ). Additionally, the difference in green and magenta area were significantly increased at 96 hours compared to 72 hours ALH ( Figure 1H ). Taken together, this indicates localized *de novo* synthesis of MDN dendritic material between 72 and 96 hours ALH, which is localized in the tips of the dendritic region. Further studies would be needed to elaborate on the biological process governing this.

Understanding the neural mechanisms underlying specific behaviors in *Drosophila melanogaster* , such as locomotion, mating, and aggression, relies heavily on the precise characterization of their governing neuronal circuits. While genetic manipulations have successfully identified the neurons critical to these behaviors, defining their morphological and developmental dynamics *in vivo* has remained difficult. The data presented here demonstrates a novel way to use the photoconvertible protein TdEOS to bridge this gap. By offering a straightforward protocol, simplified genetic requirements, robust tissue viability, and minimal photobleaching, it provides a highly accessible alternative to traditional live-cell imaging. Ultimately, these advantages establish TdEOS as a powerful and practical tool for capturing neuronal morphology across critical developmental timepoints. Although the data is not shown, we saw photoconversion in the axonal region of the MDN as well, suggesting that this protocol would be equally effective at measuring axonal dynamics overtime. Given the evolution of the GAL4-UAS system in *Drosophila,* we also believe that this technique can be utilized for studying any individual neuron or glia in larval or adults.

## Methods

*Fly husbandry*

Flies were either from crosses between the larval MDN-Gal4 driver and myristoylated TdEOS or microtubule TdEOS. All comparisons between groups were based on studies with flies grown, handled, and tested together at 25°C.

*Fly genetic reagents:*

Myristoylated TdEOS - UAS-myr::TdEOS (attp40) (BDSC# 32227)

Microtubule TdEOS - UAS-alphaTub84B.tdEOS (attp40) (BDSC# 51313)

Larval MDN-Gal4 driver - SS01613-Gal4 (GMR22H02_AD attp40; GMR23E07_DBD attp2) (Carreira-Rosario et al., 2018)

*TdEOS protocol*

Flies were reared in a dark box to prevent light exposure. After a two-day accumulation, embryos were collected overnight. The next day, newly hatched larvae were collected within a two hour window. These larvae were transferred to small containers of standard fly food and reared in a dark box. At 72 hours after larval hatching (ALH), larvae were transferred to an 3% agar surface, with no food and a mesh lid secured with parafilm; ensuring both air flow and that no larvae escape. The 49 mm agar cap with about 30 larvae was put under a stereoscope microscope (AXIO Zoom.V16) and the larvae were exposed to the DAPI 405 nm light for 20 minutes. If done correctly, this will photoconvert the GFP signal to red, although the GFP expression will be maintained. After the exposure, half of the larvae were left on the agar cap so they could be dissected, while the other half transferred back to standard food to be dissected at a later timepoint – these larvae were continuously reared in the dark.

The central nervous systems (brain and ventral nerve cord) from two larvae were dissected, handled and imaged together. The larvae were dissected in Hemolymph-Like buffer 3 (HL3) and mounted on Poly-L-Lysine Cellware 12mm round coverslips (Corning 354085). Prolong Glass Antifade Mountant 2mL (Invitrogen P36982) was used as a mounting medium and the samples were immediately imaged on a Zeiss 900 AiryScan microscope.

*Image Processing*

For color blind people, the red signal was changed to magenta for visualization.

*Quantification of dendrite area*

Using Fiji (Schindelin et al., 2012), the polygon selections tool was used to trace each dendrite individually, and the area was recorded. For each dendritic hemi-segment, the area of each channel was calculated on three slices using the measure tool. The slices selected were the middle slice of the dendritic region, and then one slice anterior and posterior. The average of the 3 slices for each channel was calculated. Then, the average areas for each channel (average magenta area and average green area) per dendrite were quantified by dividing green average area by magenta average area to get a ratio and subtracting green average area by red average area to get an absolute difference.

*Statistics*

T-tests were performed using GraphPad Prism 10.
